# The effects of the neurotoxin DSP4 on spatial learning and memory in Wistar rats

**DOI:** 10.1007/s12402-012-0076-4

**Published:** 2012-05-15

**Authors:** Joachim Hauser, Thomas A. Sontag, Oliver Tucha, Klaus W. Lange

**Affiliations:** 1 Department of Experimental Psychology, University of Regensburg, 93040 Regensburg, Germany; 2 Department of Clinical and Developmental Neuropsychology, University of Groningen, Groningen, The Netherlands

**Keywords:** Spatial memory, Reference memory, Working memory, Cogitat holeboard, DSP4, Animal model, Attention deficit hyperactivity disorder, Rat

## Abstract

The aim of the present study was to investigate the effect of DSP4-induced noradrenaline depletion on learning and memory in a spatial memory paradigm (holeboard). Since Harro et al. Brain Res 976:209–216 (2003) have demonstrated that short-term effects of DSP4 administration include both noradrenaline depletion and changes in dopamine and its metabolites—with the latter vanishing within 4 weeks after the neurotoxic lesion—the behavioural effects observed immediately after DSP4 administration cannot solely be related to noradrenaline. In the present study, spatial learning, reference memory and working memory were therefore assessed 5–10 weeks after DSP4 administration. Our results suggest that the administration of DSP4 did not lead to changes in spatial learning and memory when behavioural assessment was performed after a minimum of 5 weeks following DSP4. This lack of changes in spatial behaviour suggests that the role of noradrenaline regarding these functions may be limited. Future studies will therefore have to take into account the time-course of neurotransmitter alterations and behavioural changes following DSP4 administration.

## Introduction

Attention deficit hyperactivity disorder (ADHD) is one of the most common psychiatric disorders of childhood and adolescence and is characterised by the core symptoms hyperactivity, inattentiveness, impulsivity and distractibility; other cognitive impairments (Barkley 2006; Biederman and Faraone 2005; Heal et al. 2008; Lange et al. 2007, 2010; Tucha and Lange 2001; Tucha et al. 2006, 2008) including spatial working memory (e.g. Mills et al. 2012; Myatchin et al. 2012) may also occur. This complex behavioural and cognitive disorder affects approximately 2–7 % of children and adolescents and persists into adulthood in about 50 % of cases (Barkley 2006; Döpfner 1999). In childhood, ADHD occurs approximately three times more commonly in boys than girls (Barkley 2006; Biederman and Faraone 2005; Biederman et al. 1994; Heal et al. 2008), while the male-to-female ratio is about equal in adults (Biederman et al. 1994).

Genetic, neurobiological, social and environmental aspects have been discussed as to the aetiology and pathogenesis of ADHD (Barkley 2006; Biederman et al. 1992, 1995; Döpfner 1999). However, these approaches are still unable to sufficiently explain the aetiology of ADHD. A dysregulation of catecholaminergic neurotransmission in prefrontal cortex and its connections to striatal areas has been proposed as a major neurobiological factor (Arnsten and Dudley 2005; Heal et al. 2008; Russell et al. 2005), and the characteristic deficiency observed in ADHD has been discussed as a dysfunction of the frontostriatal system (Davids et al. 2003; Sontag et al. 2008, 2010). Dysfunctional noradrenergic and dopaminergic neurotransmission appears to be important since psychostimulants such as methylphenidate, a dopamine and noradrenaline transporter blocker, have been shown to be effective in the treatment for ADHD (Arnsten 2011). Although the specific role of dopamine and noradrenaline is as yet unclear, recent findings indicate a dysbalance between these neurotransmitters (Arnsten 2011; Heal et al. 2008).

Three dopaminergic systems have been suggested to play an important role in ADHD, that is, the mesolimbic, mesocortical and nigrostriatal pathways (for detail see Russell et al. 2005). It has been proposed that a dysfunction of dopamine in the inhibitory control of the frontal cortex is related to attentive problems and cognitive deficits and that hyperactivity/impulsivity may emerge due to impaired dopaminergic function in subcortical regions (Clements et al. 2003; Heal et al. 2008; Swanson et al. 1998).

In addition to a dysfunctional dopaminergic neurotransmission in ADHD, there is evidence suggesting that noradrenergic neurotransmission is also affected in ADHD (Arnsten 2011; Heal et al. 2008; Russell et al. 2005). While some authors have suggested low noradrenaline activity in patients with ADHD (Halperin et al. 1997; Heal et al. 2008; Oades 1987), others have proposed an increased noradrenaline activity in the prefrontal cortex of children with ADHD (Russell 2002, 2005; Solanto 1998). A role for noradrenaline in learning and memory has been elusive and controversial (e.g. Murchison et al. 2004).

A noradrenergic depletion induced by a systemic administration of the neurotoxin *N*-(2-chloroethyl)-*N*-ethyl-2-bromobenzylamine (DSP4) can be used in order to elucidate the role of noradrenaline in cognitive functions such as spatial working memory. This approach may also provide an animal model (e.g. of ADHD) with a central noradrenergic lesion. This approach allows the selective destruction of terminals of noradrenergic neurons originating in the locus coeruleus (Fritschy and Grzanna 1991) and reduces brain noradrenaline activity in a dose-dependent manner (Cheetham et al. 1996). Cognitive performance following the administration of DSP4 has been studied for various cognitive functions such as working memory and reference memory (Ohno et al. 1993, 1997; Sontag et al. 2008, 2011), short-term memory and attention (Ruotsalainen et al. 1997), discrimination learning (Al Zahrani et al. 1997) and motor activity (Jones and Hess 2003). However, while some authors observed an impaired performance in these functions, others were unable to find any significant alteration.

Since Harro et al. (2003) have demonstrated that short-term effects of DSP4 administration include both noradrenaline depletion and changes in dopamine and its metabolites—with the latter vanishing within 4 weeks after the neurotoxic lesion—the behavioural effects observed immediately after DSP4 administration cannot solely be related to noradrenaline. Previous studies assessing the behavioural effects of DSP4 were performed one or 2 weeks after DSP4 administration (Al Zahrani et al. 1997; Ohno et al. 1993,1997; Ruotsalainen et al. 1997; Sontag et al. 2008, 2011). The aim of the present study was to investigate the effect of the sole depletion of noradrenaline on learning and memory in a spatial memory paradigm. Behavioural assessment of spatial learning, reference memory and working memory was therefore performed five to 10 weeks after DSP4 administration. We have used a holeboard task where rats are required to find hidden food pellets (Heim et al. 2000).

## Methods

### Animals and feeding procedure

Forty-eight male Wistar rats (Charles River Laboratories, Sulzbach, Germany) aged 12 weeks (weight approximately 300 g at the beginning of the experiment) were used. The animals were kept in standard cages on a 12-h light/12-h dark cycle (room temperature, 22 °C; humidity, 50 %). The access to food was restricted since the learning paradigm on the Cogitat holeboard is based on food reinforcement (i.e. 45 mg dustless sucrose pellets, Bio-Serv, Frenchtown, New Jersey, USA). Water was provided ad libitum. Rats were fed (standard food pellets, Ssniff Spezialitäten GmbH, Soest, Germany) after the testing procedures for 1 h a day. The rats’ body weight and general health were carefully controlled, and a weight reduction of more than 15 % compared to freely fed rats was avoided in order to prevent stress and subsequent changes in dopaminergic neurotransmission (Bear 1999; Deroche et al. 1995; Pothos et al. 1995).

All experiments were performed in accordance with the national laws (German law on Protection of Animals) and the principles of laboratory animal care (NIH publication No. 86- 23, revised 1985).

### The Cogitat holeboard

The learning behaviour of the rats was tested with the Cogitat holeboard system (Cogitron GmbH, Göttingen, Germany). This system consists of a board with 25 holes. Each hole of the board is closed at its lower end by an adjustable feeding plate with a depression for a food pellet. Feeding plate and food pellets are of the same colour. The ground below the feeding plate is covered with food pellets, in order to prevent the animals from finding the pattern of the pellet distribution by using olfactory stimuli. Each hole is fitted with infrared light beams at different levels of the hole to measure activity. An interruption of the upper light beam is defined as an “inspection”, while the term “visits” is related to the lower light beam. Finally, there is an infrared beam at the feeding plate measuring the collection of a food pellet. A more detailed description of the Cogitat holeboard system can be found elsewhere (Heim et al. 2000). In the present experiment, eight of the 25 holes were baited. A search trial was automatically finished when a rat had found all the hidden pellets or after a fixed period of 60s.

On the Cogitat holeboard, performance of rats can be divided in reference memory and working memory. Reference memory is defined as the ability to remember the baited pattern and should improve over time. The focus is therefore on comparisons between trials. By contrast, working memory is defined as the ability to remember which holes a rat has already inspected, visited and/or emptied in one trial. In each single trial, the following parameters were measured: (1) working memory error (i.e. the percentage of inspections to previously baited holes in a single trial in relation to the total number of holes inspected) and (2) reference memory error (i.e. the percentage of inspections to nonbaited holes in relation to the total number of holes inspected).

### DSP4 administration, habituation and testing procedure

The rats were habituated for 10 days to room conditions, light/dark circle, feeding and other routine procedures. They were then randomly divided into four groups of 12 rats each. The animals of the control group were injected with saline; the animals of the other three groups received an injection of DSP4 (Sigma-Aldrich, Schnelldorf, Germany) at a dose of 10, 20 or 50 mg/kg body weight. DSP4 was dissolved in saline; both DSP4 solution and saline were administered intraperitoneally. A 5-week period followed the administration of DSP4 or saline during which body weight and health were checked once daily and the feeding procedure was less restrictive. This period also allowed for a recovery of the peripheral noradrenergic system (Fritschy and Grzanna 1991).

During the recovery period, animals were habituated to the Cogitat system as follows. Rats were placed on the holeboard for 5 min once daily for 2 weeks. During this habituation phase, eight holes were baited with a pellet and a different pattern was chosen each day in order to ascertain that each hole was baited at least once.

After habituation to the holeboard, the rats were tested once daily for 5 weeks. In this testing period, a fixed pattern of baited pellets was used. A trial was finished when the rat had found all pellets or after a fixed period of 60s. The order in which the animals were tested during habituation and testing periods was randomised in order to reduce circadian influences.

### Statistical analysis

Comparisons between the DSP4 groups were made for each week by avering the results of five consecutive days. The statistical analysis of differences between DSP4 groups was carried out using the Mann–Whitney *U* test (between-subject design); *p* values lower than 0.05 were considered statistically significant. With regard to learning performance within each group, the first week of testing was compared with the following weeks by using the Wilcoxon test; *p* values of <0.05 were considered statistically significant. All statistical analyses were carried out using the Statistical Package for Social Sciences 19.0 (SPSS) for Windows.

## Results

### Reference memory error

**Fig. 1:**
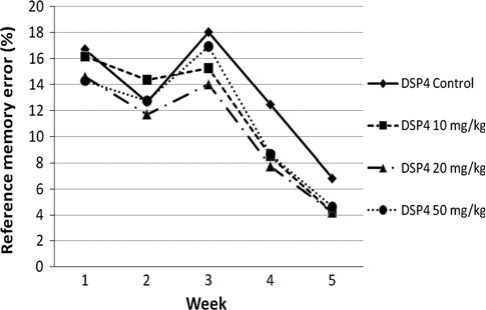
Mean percentage of reference memory errors for all groups over 5 weeks

As for spatial reference memory, the performance of the DSP4 and control groups is presented in Fig. 1 and Table 1. Most of the comparisons between the DSP4 and saline-treated groups did not reach statistical significance, except the following: in the second week, the DSP4_10 mg/kg group made significantly more reference memory errors than the DSP4_20 mg/kg group (*p* = 0.046; *z* = −1.992). In the fourth week, the control group made significantly more reference memory errors compared to the DSP4_20 mg/kg group (*p* = 0.04; *z* = −2.05).

**Table 1:** Performance on the Cogitat holeboard of the DSP4 and saline groups for each week (mean ± standard error)

	Control	DSP4_10 mg	DSP4_20 mg	DSP4_50 mg
Reference memory error				
Week 1	16.71 ± 2.49	16.15 ± 2.14	14.61 ± 2.64	14.28 ± 1.82
Week 2	12.64 ± 1.27	14.35 ± 1.25	11.71 ± 1.87^b^	12.74 ± 2.44
Week 3	18.03 ± 2.4	15.28 ± 1.93	14.02 ± 1.66	16.95 ± 1.94
Week 4	12.45 ± 1.82	8.43 ± 1.69^1^	7.73 ± 1.19^a1^	8.63 ± 1.5^1^
Week 5	6.80 ± 1.35^1^	4.20 ± 1.15^1^	4.09 ± 1.22^1^	4.71 ± 1.22^1^
Working memory error				
Week 1	11.84 ± 1.06	10.58 ± 2.01	12.54 ± 0.93	14.52 ± 1.2^a^
Week 2	10.14 ± 1.48	7.60 ± 0.93	8.04 ± 1.24^1^	9.41 ± 1.88^1^
Week 3	7.04 ± 1.27^1^	7.79 ± 0.94	7.12 ± 1.49^1^	7.76 ± 0.74^1^
Week 4	6.32 ± 1.02^1^	7.47 ± 2.1	7.07 ± 1.48^1^	6.15 ± 1.25^1^
Week 5	7.11 ± 1.08^1^	6.69 ± 1.56^1^	4.49 ± 1.01^1^	5.49 ± 1.32^1^

^a^Compared with control group, ^b^ compared with DSP4_10 mg/kg, ^1^ compared with week 1, ^a, b, 1^: *p* ≤ 0.05

The control group made significantly fewer reference memory errors in week 5 than in week 1 (*p* = 0.005; *z* = −2.824); the other comparisons were not statistically significant. The DSP4_10 mg/kg animals made significantly fewer reference memory errors in the last 2 weeks than in the first week (*p* = 0.01; *z* = −2.589 compared with week 4; *p* = 0.003; *z* = −2.981 in comparison with week 5); the other comparisons did not reach statistical significance. The dose of 20 mg/kg DSP4 caused significantly fewer reference memory errors in week 4 (*p* = 0.034; *z* = −2.118) and week 5 (*p* = 0.004; *z* = −2.903) than in week 1. The rats treated with the high dose of DSP4 (50 mg/kg) made significantly more reference memory errors in the first week than in the fourth (*p* = 0.008; *z* = −2.667) and fifth (*p* = 0.002; *z* = −3.059) weeks. The other comparisons were not statistically significant.

### Working memory error

**Fig. 2:**
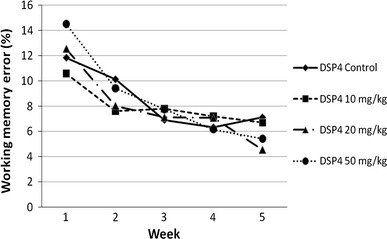
Mean percentage of working memory errors for all groups over 5 weeks

With regard to the working memory error, the spatial learning performance of the DSP4 and control groups is presented in Fig. 2 (for more detailed data see Table 1). The administration of 50 mg/kg DSP4 significantly increased the working memory error compared with control animals (*p* = 0.046; *z* = −1.992). None of the remaining comparisons between DSP4 groups or the saline-treated group achieved statistical significance.

The animals of the control group made significantly more working memory errors in the first week than in week 3 (*p* = 0.034; *z* = −2.119), week 4 (*p* = 0.015; *z* = −2.432) and week 5 (*p* = 0.019; *z* = −2.353). The DSP4_10 mg/kg rats made in the last week only significantly fewer working memory errors than in the first week (*p* = 0.012; *z* = −2.51); all other comparisons between week 1 and the following weeks were not statistically significant. The animals treated with the medium dose of DSP4 (20 mg/kg) showed significantly fewer working memory errors in the first week than in all the following weeks (compared with week 2: *p* = 0.008; *z* = −2.667; compared with week 3: *p* = 0.008; *z* = −2.667; compared with week 4: *p* = 0.019; *z* = −2.353; compared with week 5: *p* = 0.005; *z* = −2.824). Similar to the DSP4_20 mg/kg group, the DSP4_50 mg/kg group made significantly more working memory errors in the first week than in the second week (*p* = 0.041; *z* = −2.04), the third week (*p* = 0.002; *z* = −3.059), the fourth week (*p* = 0.002; *z* = −3.059) and the fifth week (*p* = 0.004; *z* = −2.903).

## Discussion

Several previous studies have presented evidence of impaired cognitive functioning following DSP4 administration (Compton et al. 1995; Wenk et al. 1987). However, some authors were unable to demonstrate any impairments (Al Zahrani et al. 1997; Benloucif et al. 1995; Langlais et al. 1993). All previous studies discussed in this paper share two important aspects, that is, (1) they use a DSP4 dose of 50 mg/kg only and (2) they assume that the treatment with DSP4 affects the noradrenergic system exclusively, independent of the time elapsed following the administration of DSP4 (Cheetham et al. 1996; Fritschy and Grzanna 1991). By contrast, Harro et al. (2003) demonstrated that the effect following DSP4 administration changes over time and that the dopaminergic system is also affected after DSP4 administration. However, the latter effect appears to be temporary and to vanish within a few weeks. The aim of the present study was to examine the exclusive effect of a noradrenergic depletion by DSP4 on cognitive skills such as learning, reference memory and working memory in a spatial memory task. Behavioural testing was therefore performed 5 weeks after DSP4 administration, when dopaminergic effects of DSP4 have been reported to be greatly diminished (Harro et al. 2003).

All groups showed clear improvements in the Cogitat holeboard paradigm, that is, the rats of all groups were able to enhance their performance in spatial learning and memory over time. In detail, all DSP4 groups and the control group displayed significant improvements in working memory over the course of 5 weeks. With regard to reference memory error, all groups displayed a moderate improvement in the second week followed by an increase in errors in the third week; the percentage of reference memory errors of the DSP4_50 mg and control groups exceeded the values of the previous weeks. In the remaining weeks, a consistent and statistically significant decline of reference memory error could be observed. These data suggest that all groups were able to learn the paradigm and to improve their performance over time, as shown by a clear reduction in both working memory and reference memory errors.

Another aim of the present study was to investigate the effect of central noradrenergic depletion on rats’ performance in a spatial memory task as assessed by comparing various DSP4 doses and a saline-treated group. Taken together, the present data suggest little difference in the performance of spatial memory between the DSP4 groups and the control group. A limitation of the present study is the lack of histological or neurochemical analyses regarding the central noradrenergic system.

With regard to reference memory error, only two comparisons were statistically significant (i.e. the comparisons between DSP4_10 mg/kg and DSP4_20 mg/kg in week 2 and between DSP4_20 mg/kg and controls in week 4). Over the course of time, the DSP4_50 mg/kg group always made fewer reference memory errors than the saline-treated animals. The difference between the medium and low doses of DSP4 in week 2 as well as the difference in DSP4 between animals that received medium dose and control animals reached statistical significance. Interestingly, the low dose in the first comparison mentioned and the control group in the latter comparison showed more reference memory errors than the medium dose or high dose, suggesting a beneficial effect of DSP4. This seems to be counterintuitive and should be interpreted as an artefact, not least because there does not appear to be a linear relationship between DSP4 dose, more precisely the dose-dependent noradrenergic depletion as suggested by Cheetham et al. (1996), and the performance in spatial reference memory. In summary, our data indicate that the DSP4 administration had a minor effect on reference memory, which is in accordance with previous findings (Ohno et al. 1993, 1997).

As to spatial working memory, in the first week, the DSP4_50 mg/kg group made significantly more working memory errors compared to the control group. In subsequent weeks, the DSP4_50 mg/kg animals showed fewer working memory errors than controls, with the exception of week 4, where the group treated with the high dose displayed slightly more errors. This is in contrast to previous publications reporting working memory impairments (Ohno et al. 1993; Sontag et al. 2008). Our data suggest no linear relationship between noradrenaline depletion induced by DSP4 and the spatial working memory performance as assessed with the Cogitat holeboard. In the present experiment, the spatial working memory of rats was not affected by DSP4 administration. This result is not in line with previous publications (Ohno et al. 1993; Sontag et al. 2008) but agrees with other studies that were unable to reveal an effect of DSP4 on various cognitive functions (Al Zahrani et al. 1997; Benloucif et al. 1995; Langlais et al. 1993) including spatial working memory (Sontag et al. 2011).

In summary, the present study does not support the notion that noradrenergic depletion following DSP4 administration affects the spatial memory skills of rats in a holeboard paradigm such as Cogitat. Neither was the ability to learn a certain pattern affected by DSP4 nor were there any substantial or systematic differences between the DSP4 and saline-treated groups with regard to spatial reference memory and spatial working memory.

Attempts at the explanation of the present findings will remain speculative. It is conceivable that the DSP4-induced noradrenaline depletion is limited over time. However, there is no support in the literature for a time-limited effect of DSP4 on noradrenaline, at least not for the high dose (50 mg/kg) (Harro et al. 2003). On the contrary, the lasting effect of DSP4 on noradrenergic terminals has been described as one of the advantages of DSP4 administration (Cheetham et al. 1996; Fritschy and Grzanna 1991), and there has been no indication for a time-limited effect (Al Zahrani et al. 1997; Benloucif et al. 1995; Cheetham et al. 1996; Compton et al. 1995; Fritschy and Grzanna 1991; Langlais et al. 1993; Ohno et al. 1993; Wenk et al. 1987). One could also conclude that the paradigm used in the present experiment (holeboard system) did not test cognitive functions affected by noradrenergic depletion or that the task was not sensitive enough to reveal existing effects. However, the following observations do not support this view. First, there is evidence that working memory is modulated by catecholamines and noradrenaline in particular (Ohno et al. 1993; Ramos and Arnsten 2007; Sontag et al. 2008). Second, Sontag et al. (2008), using the Cogitat holeboard, have shown that noradrenergic depletion by the administration of DSP4 causes significant impairment in working memory without affecting reference memory. In contrast to the present study, the authors’ focus was not on the effect of DSP4 on learning but rather on the effect of noradrenaline depletion on a task that rats had previously learnt (Sontag et al. 2008, 2011). In the studies by Sontag et al. (2008, 2011), rats were trained first, then treated with DSP4 and tested after a 2-week recovery of the peripheral noradrenaline system, as has been suggested by several studies using DSP4 (Al Zahrani et al. 1997; Ohno et al. 1993; Ruotsalainen et al. 1997). Harro et al. (2003) have shown that DSP4 does not solely affect noradrenaline terminals, but dopamine and serotonin levels as well as dopamine receptor concentrations may also be affected (Harro et al. 2003). In conclusion, other neurotransmitters apart from noradrenaline may be directly or indirectly influenced by DSP4, as mentioned by Sontag et al. (2008), and this influence may be time-limited, as suggested by Harro et al. (2003). This may be an explanation for the differing findings of Sontag et al. (2008) and the present study.

Another explanation for the present findings is that there may be no direct functional relationship between central noradrenaline level and spatial memory. Noradrenaline may not affect spatial memory but rather other more fundamental cognitive functions such as attention. However, there is evidence disagreeing with this viewpoint (see Ohno et al. 1993; Sontag et al. 2008). In conclusion, the administration of DSP4 did not lead to changes in spatial learning and memory when behavioural assessment was performed 5 weeks after DSP4 administration.
